# Problems of equity in US HIV integrated planning, 2015–2021: Enacting a bounded justice continuum

**DOI:** 10.1177/13634593251375041

**Published:** 2025-11-05

**Authors:** Stephen Molldrem, Nivan Wadhawan, Alec Manning, Justin D. Edwards

**Affiliations:** 1 The University of Texas Medical Branch, USA

**Keywords:** critical policy studies, HIV policy, healthcare reform, integrated planning, justice

## Abstract

The US HIV/AIDS response is notably worse than those of other high-income countries. The country’s epidemic is marked by low viral suppression rates, high incidence, lacking coordination, and entrenched disparities along lines of sexuality, race/ethnicity, gender, class, and other factors. In 2010, the *National HIV/AIDS Strategy for the United States* (*NHAS*) was launched, centering an equity-oriented vision that prioritized marginalized groups. *NHAS* implementation required states to create HIV integrated plans to better coordinate services and meet populations’ needs. We used Carol Bacchi’s “What’s the Problem Represented to Be?” approach to analyze 20 jurisdictions’ plans, focusing on how they incorporated equity-oriented principles articulated in *NHAS*’s vision statement and other factors such as plans for integration across HIV care, surveillance, and prevention programs. Building on Melissa Creary’s concept of “bounded justice,” we show that integrated plans enacted a “bounded justice continuum,” wherein some states pursued more equity-oriented strategies than others. We argue that this reflects constraints planners faced and the structure of US federalism, where implementing jurisdictions operated in variously restrictive or enabling conditions related to state-level politics, available public health infrastructure, and other factors. Our approach and the bounded justice continuum concept can be useful for scholars studying the rollout of equity-oriented policies in federal systems where local implementations will vary widely. We ultimately arrive at a positive assessment of US HIV integrated planning. However, we also advocate for more transformative reforms to ensure that people living with and affected by HIV can access universal healthcare, social services, housing, and employment.

## Introduction

In 2010, the United States (US) Office of National AIDS Policy (ONAP) launched the *National HIV/AIDS Strategy for the United States* (*NHAS*; ONAP, 2015).^1^ *NHAS* was billed as the “first comprehensive” federal plan to address the country’s HIV/AIDS epidemic (The White House, 2015), which has long been the worst-managed among high-income countries (Kaiser Family Foundation, 2025; Sullivan et al., 2021). The plan laid out strategic goals for the country to reduce new HIV infections and retain people living with HIV (PLHIV) in care. *NHAS* was notable for its focus on improving health equity for historically marginalized groups affected by HIV/AIDS. Its vision statement asserted: “The United States will become a place where new HIV infections are rare, and when they do occur, every person, regardless of age, gender, race/ethnicity, sexual orientation, gender identity, or socio-economic circumstance, will have unfettered access to high quality, life-extending care, free from stigma and discrimination” (ONAP, 2015). The explicit naming of minoritized populations was notable at a time when many such groups – particularly sexual and gender minorities – had been largely excluded from federal health policy paradigms. In 2010, this had begun to change under the Obama administration, when sexual and gender minorities were often explicitly included in federal initiatives (Stewart-Winter, 2018).

As part of *NHAS* implementation, in 2015, the US Centers for Disease Control and Prevention (CDC) and Health Resources and Services Administration (HRSA) released HIV integrated planning guidance for states (Centers for Disease Control and Prevention (CDC) and Health Resources and Services Administration (HRSA), 2015). Integrated planning was one strategy to align the country’s fragmented response across CDC-funded HIV surveillance and prevention programs and HRSA-funded care and social service programs. States were required to create integrated plans covering 2017–2021. Planning processes were required to engage key health system stakeholders and representatives of key populations to produce goals corresponding to *NHAS*.

This paper reports results from an analysis of 20 HIV integrated plans created under the 2015 guidance, focusing on how well plans aligned with the *NHAS* vision. We first describe the history of *NHAS* and how integrated planning efforts were framed as a path toward achieving greater equity. We then describe the theories and methods we used to analyze the plans and present our findings. Our analysis focused on how plans framed key issues related to health equity in four ways: (1) Inclusion of key populations in programs; (2) Integration of HIV programs across the domains of HIV care, surveillance, and prevention; (3) Integration of HIV data infrastructures from different program domains; and (4) Inclusion of key populations and stakeholder groups in HIV integrated planning processes. We build on Creary’s (2021) notion of “bounded justice” to argue that HIV integrated planning enacted a “bounded justice continuum,” wherein some states pursued more rigorously equity and justice-oriented plans than others within varying material, political, and other constraints. Differences across HIV integrated plans were deeply shaped by the structure of US federalism, which gives states broad leeway in determining how federal policies are implemented, thus affecting possibilities for justice-oriented outcomes.

We ultimately arrive at a positive assessment of how HIV integrated planning required agencies to collaboratively work to improve states’ HIV responses. However, we also conclude that bringing meaningful closure to the epidemic will require deeper social transformation that cannot be addressed by integrated planning or the US healthcare system and economy as presently structured. We thus advocate for more transformative reforms to ensure that people affected by HIV can access universal healthcare, services, housing, and employment. We also argue that the structure of our research questions and the “bounded justice continuum” framework can be useful for scholars conducting health equity and justice-oriented impact assessments and related empirical studies of health policy implementations in federal systems like the US.

## Background

The US did not have a comprehensive domestic HIV/AIDS strategy until *NHAS* in 2010, and state-level integrated HIV planning was not required until 2015 (CDC and HRSA, 2015; ONAP, 2015; Yehia and Frank, 2011). The purpose of *NHAS* and integrated planning was to improve the national response by re-orienting it around *NHAS*’s equity-focused vision and associated goals.

The history of the US HIV response is one of fragmentation and atomization, rather than integration and coordination (Fairchild and Bayer, 2011; Molldrem, 2020; Sweeney et al., 2013). Different HIV programs – particularly care, prevention, and surveillance – have been managed in separate programs, through distinct funding streams, and using siloed data infrastructures. Each of the three key HIV domains have historically been organizationally rather separate, owing largely to privacy and stigma-related concerns around data sharing that resulted in advocacy against names-based reporting and expanded public health data uses (Fairchild et al., 2007; Molldrem and Smith, 2020). However, particularly since the emergence of effective HIV treatments in 1996, the US Department of Health and Human Services has pushed for more granular names-based HIV case reporting and for the integration of HIV care, prevention, and surveillance data infrastructures and programs (Fairchild and Bayer, 2011). This work has been pursued to enact a more coordinated response through programmatic integration, and has intensified with developments such as the nationwide implementation of names-based HIV case reporting in 2008 and the emergence of knowledge that HIV treatment also prevents transmission (Cohen et al., 2014; Fairchild and Bayer, 2011; Molldrem and Smith, 2020; Satcher Johnson et al., 2024).

HIV integrated planning arose at a particular moment in the history of three interlinked areas: (1) Obama-era healthcare reforms designed to expand access to care, particularly the Patient Protection and Affordable Care Act of 2010 (ACA); (2) the emergence of digital health infrastructures; and (3) changes in the clinical and public health strategies used to manage HIV/AIDS that emphasized programmatic and infrastructural integration to improve outcomes (Molldrem and Smith, 2020; Satcher Johnson et al., 2024). A discussion of these factors illuminates the shifting conditions of possibility that gave rise to HIV integrated planning post-2015.

The first development was the Obama administration’s healthcare reform strategy, pursued early in the administration with support from a broad-based liberal-progressive coalition (Dawes, 2016). Passage of the ACA in 2010 was the cornerstone of these efforts, particularly through establishing state-level insurance exchanges, individual coverage mandates, and funding for states to expand Medicaid (health insurance for low-income individuals and families). ACA implementation was accompanied by ancillary efforts to advance health equity, and HIV/AIDS was one area of focus for the coalition that aided the ACA’s passage and implementation. One key initiative was *NHAS*, which launched in 2010 alongside a federal implementation plan explicitly designed to work synergistically with ACA implementation (ONAP, 2015).

The second key factor that motivated HIV integrated planning was the digitization of the healthcare system, which accompanied ACA reforms. In the 2010s, the US transitioned from paper-based medical records to electronic health records and digital health systems that federal agencies such as the Centers for Medicare and Medicaid Services and Office of the National Coordinator for Health IT (ONC) required states and healthcare organizations to adopt (ONC, 2015). Subsequent initiatives such as CDC’s Data Modernization Initiative and the 21st Century Cures Act of 2016 encouraged or required organizations to make their data systems able to exchange health data more easily to coordinate care, public health action, research, and other functions. These digital transformations in US healthcare laid the groundwork for a more interoperable health data ecology, including in HIV (Cohen et al., 2014; Satcher Johnson et al., 2024).

The third factor that compelled HIV integrated planning in the US was the emergence of HIV treatment-as-prevention. During the mid-2010s, scientific consensus emerged around the fact that PLHIV who are taking antiretroviral treatment and virally suppressed cannot transmit HIV via sexual contact (Rodger et al., 2016). In the 2010s, federal policy documents involved in the implementation of *NHAS* and related programs such as the “HIV Care Continuum Initiative” noted the greatly decreased risk of HIV transmission by PLHIV who were in treatment and virally suppressed (ONAP, 2015), and treatment-as-prevention was formally adopted as federal policy in 2017 (McCray and Mermin, 2017). The shift toward treatment-as-prevention as a public health strategy created added incentives for health systems to keep PLHIV retained in care and to use data to re-link people to care who had fallen out of care (Cohen et al., 2014; Molldrem, 2020; Molldrem and Smith, 2020; Satcher Johnson et al., 2024).

This backdrop shaped the push for state-level HIV integrated planning as part of *NHAS* implementation. Partly because of the liberal-progressive political orientation of the Obama administration, *NHAS* aimed to improve outcomes for disparity groups, which were explicitly named in the *NHAS* vision statement and associated goals, to which state-level integrated plans were required to correspond (CDC and HRSA, 2015). Our study sought to understand how states pursued HIV integrated planning in the context of *NHAS*’s commitment to equity for marginalized populations and against the backdrop of larger structural transformations related to healthcare reform, digitization, and treatment-as-prevention.

## Theoretical frameworks, methods, and materials

We used Carol Bacchi’s “What’s the Problem Represented to Be?” (WPR) critical policy analysis approach to analyze the role of HIV integrated planning in the US HIV response and *NHAS* implementation (Bacchi, 2012; Bacchi and Goodwin, 2016). WPR involves answering six questions to determine how social problems are constructed and framed by policymakers. We used a WPR worksheet to answer the six questions, describing how HIV integrated planning was framed by the federal integrated planning guidance in the context of broader US healthcare reform efforts, digitization, treatment-as-prevention, and *NHAS* rollout. We determined that integrated planning was put forward as a partial solution to the “problem” of an uncoordinated national HIV response. This problem was itself framed as one effect of the country’s overall failure to effectively manage its HIV/AIDS epidemic, with targets such as new HIV incidence and viral suppression rates falling short of international goals. Table 1 shows the six questions and our answers in the WPR worksheet.

**Table 1.** WPR worksheet questions and answers for US HIV integrated planning.

**Question 1:** What’s the “problem” represented to be in a specific policy or policies?
**Answer 1a:** The problem is the poor and inequitable response to HIV in the US, which falls far below international benchmarks and is the worst among high-income countries.
**Answer 1b:** The more specific policy problem is the lack of a coordinated or integrated response that is attentive to key populations affected by HIV/AIDS.
**Question 2:** What deep-seated presuppositions or assumptions underlie this representation of the “problem”?
**Answer 2:** The deep-seated presuppositions and assumptions underlying the representation are (1) that the poor US HIV response is the result of a lack of programmatic integration and (2) that better integration and coordination will lead to improved outcomes.
**Question 3:** How has this representation of the “problem” come about?
**Answer 3:** The representation of the US HIV response as suffering because of lacking coordination and integration to address key populations’ needs came about because of the priorities of the liberal-progressive healthcare reform coalition that formed in support of the Obama administration’s goals, which advocated for the inclusion of key groups in *NHAS* and favored the integration of programs to address key disparities.
**Question 4:** What is left unproblematic in this problem representation? Where are the silences? Can the “problem” be conceptualized differently?
**Answer 4:** Integrated planning and the integration of programs around meeting the needs of key populations affected by HIV/AIDS cannot solve much deeper underlying social inequities in the US. The “problem” of the poor US HIV response could have been represented as stemming from a lack of universal healthcare, guaranteed housing, and jobs.
**Question 5:** What effects (discursive, subjectification, lived) are produced by this representation of the “problem”?
**Answer 5:** One major effect produced by representing the problem of a poor and uncoordinated US HIV response was the call to integrate HIV programs at the jurisdictional level to better address HIV-related disparities.
**Question 6:** How and where has this representation of the “problem” been produced, disseminated and defended? How has it been and/or how can it be disrupted and replaced?
**Answer 6:** The problem of an uncoordinated and inequitable HIV response was represented in *NHAS* and 2015 federal HIV integrated planning guidance, and then in integrated plans themselves. This representation of the problem can be disrupted and replaced by promoting broad-scale social transformation that would guarantee healthcare, jobs, housing, and other basic necessities. However, our main goal was to understand how the integrated planning paradigm was enacted.

Source: Adapted from Bacchi and Goodwin (2016: 20).

Our WPR analysis led us to construct four research questions (**RQ**s). **RQ1** focused on *key populations* named in the *NHAS* vision, along with one sub-question about intersectionality and five sub-questions about specific named groups (**RQ**s 1a–1f). **RQ2** focused on *program integration*. **RQ3** focused on *data systems*. **RQ4** focused on states’ *integrated planning process*. Table 2 shows the **RQ**s.

**Table 2.** Research questions used to analyze HIV integrated plans.

**RQ1, on *the inclusion of key populations*:** “How do the integrated plans represent key populations named in the *NHAS* vision statement?”
**RQ1a:** “How do the integrated plans represent intersectional populations (i.e. who experience multiple forms of marginalization) or use the language of intersectionality?”
**RQ1b:** “How do the integrated plans represent key populations by sexual orientation?”
**RQ1c:** “How do the integrated plans represent key populations by gender?”
**RQ1d:** “How do the integrated plans represent key populations by race and ethnicity?”
**RQ1e:** “How do the integrated plans represent key populations by class or socio-economic circumstance?”
**RQ1f:** “How do the integrated plans represent key populations by age?”
**RQ2, on *program integration:*** “How do the integrated plans propose to integrate programmatic activities from care, surveillance, and prevention?”
**RQ3, on *data system integration:*** “How do the integrated plans propose to integrate HIV data systems from care, surveillance, and prevention?”
**RQ4, on states’ *integrated planning process:*** “How did states describe their integrated planning process, including key stakeholders involved and methods used?”

**Figure 1.**
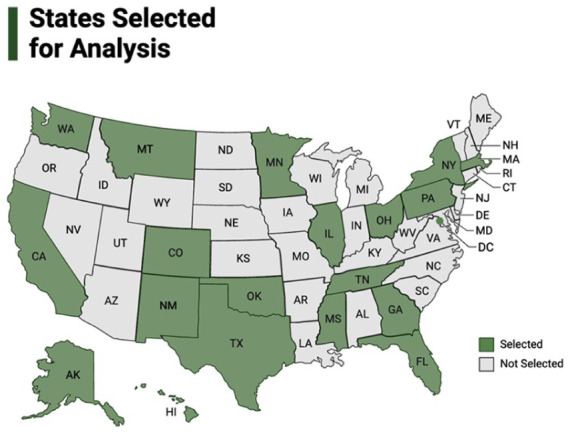
States selected for analysis.

We gathered state HIV integrated plans created under the 2015 CDC/HRSA guidance. Most plans were housed on a central website; we emailed several states to secure copies of their plans. We selected 20 jurisdictions (19 states and the District of Columbia, herein “states”) to analyze. We constructed a balanced sample across regions, demographics, and differing epidemiologic profiles. We considered HIV incidence, whether states had expanded Medicaid under the ACA, racial/ethnic demographics, HIV prevalence rates, as well as more qualitative metrics such as whether the state had more than one large urban area or jurisdictions included in the *Ending the HIV Epidemic* initiative launched in 2019. States selected are shown in Figure 1. Table 3 shows data corresponding to key selection criteria for each state, including state population demographics, HIV prevalence, and racial disparities in HIV incidence.

**Table 3.** Key state selection criteria.

State	Medicaid expansion by 2015?^a^	Estimated state population (2015)^b^	% Of state living in poverty^c^	HIV incidence per 100k^d^	% State residents White alone^e^	% PLHIV White^d^	% PLHIV Black^d^	% PLHIV Hispanic/Latino^d^	% PLHIV Asian^d^	% PLHIV American Indian or Alaska Native^d^	% PLHIV Native Hawaiian or other Pacific Islander^d^	% PLHIV multiple races^d^
Alaska	Yes (2015)	737,498	10.4	106.2	59.4	45.68	12.56	9.89	3.30	23.23	0.31	5.02
California	Yes (2014)	38,918,045	15.4	371.0	41.2	40.79	17.60	34.95	3.80	0.30	0.22	2.34
Colorado	Yes (2014)	5,450,623	11.5	251.5	70.7	61.49	14.30	20.92	0.96	0.54	0.12	1.67
DC	Yes (2014)	675,400	17.7	2665.0	39.6	16.74	72.45	7.18	0.52	0.09	0.05	2.97
Florida	No	20,209,042	15.8	609.0	57.7	28.52	46.83	22.02	0.41	0.06	0.03	2.13
Georgia	No	10,178,447	17.2	564.0	51.9	19.74	68.84	6.52	0.43	0.05	0.03	4.39
Hawaii	Yes (2014)	1,422,052	10.7	230.0	22.9	51.04	5.33	11.09	14.89	0.29	8.57	8.79
Illinois	Yes (2014)	12,858,913	13.6	324.0	61.4	28.38	46.78	18.29	1.16	0.10	0.06	5.23
Massachusetts	Yes (2014)	6,794,228	11.5	333.9	69.6	41.23	28.92	26.10	1.86	0.11	0.02	1.76
Minnesota	Yes (2014)	5,482,032	10.2	166.6	77.5	49.53	34.43	9.62	1.74	1.48	0.07	3.14
Mississippi	No	2,988,471	22.1	363.8	56.0	19.84	72.79	3.13	0.14	0.08	0.01	4.01
Montana	No (2016)	1,030,475	14.4	63.7	84.5	80.84	3.28	5.11	0.18	5.47	0.37	4.75
New Mexico	Yes (2014)	2,089,291	19.8	179.7	51.0	35.98	6.11	47.09	0.42	7.66	0.06	2.68
New York	Yes (2014)	19,654,666	15.5	781.6	55.2	19.24	39.76	33.66	1.30	0.06	0.01	5.97
Ohio	Yes (2014)	11,617,527	14.8	204.6	77.0	46.05	43.93	6.00	0.42	0.08	0.01	3.52
Oklahoma	No (2021)	3,909,500	16.0	176.0	63.5	56.39	23.32	8.96	0.95	5.12	0.12	5.16
Pennsylvania	Yes (2015)	12,784,826	13.1	308.6	75.0	30.18	48.38	16.24	0.69	0.09	0.07	4.36
Tennessee	No	6,591,170	16.7	295.0	72.2	36.18	55.53	4.89	0.35	0.05	0.04	2.96
Texas	No	27,470,056	15.9	357.1	50.1	27.14	36.92	31.25	0.93	0.05	0.02	3.69
Washington	Yes (2014)	7,163,657	12.2	203.7	66.6	63.13	15.52	13.31	3.23	1.10	0.47	3.25

a Medicaid expansion data are drawn from the Kaiser Family Foundation (KFF): https://www.kff.org/status-of-state-medicaid-expansion-decisions/.

b State population estimates for 2015 are drawn US census data: https://www.census.gov/data/tables/time-series/demo/popest/2010s-state-detail.html.

c State poverty data for 2015 come from US Census state profiles: https://www.census.gov/data/datasets/2015/demo/saipe/2015-state-and-county.html.

d HIV prevalence data for 2015 are drawn from the 2015 CDC US HIV Surveillance Report, Table 24, rounded to the second decimal: https://web.archive.org/web/20250116042009/https://www.cdc.gov/hiv/pdf/library/reports/surveillance/cdc-hiv-surveillance-report-2015-vol-27.pdf.

e State rates of individuals identifying as “white alone” are drawn from US census 2020 state profiles: https://www.census.gov/library/stories/state-by-state.html.

Following Bacchi’s (2012) approach, team members used critical textual reading and group dialog to analyze all 20 plans. We worked through and discussed one-to-three per week as a group. Individual team members summarized findings for each RQ in a shared spreadsheet with tabs for each analyst’s findings and one tab for synthesizing all analysts’ findings within each RQ. The team also kept shared notes cataloging group discussions.

### Using bounded justice to theorize HIV integrated planning

Drawing on Creary (2021), we came to understand the federal HIV integrated planning paradigm as an attempt to enact a kind of “bounded justice” particular to Obama-era liberal-progressive politics, which emphasized health equity and policy reform. For Creary, “Bounded justice describes the inherent limitations of even forward-thinking and justice-based notions of inclusions. It is a concept and analytic that reveals how inclusive programs and policies often fail to recognize the fundamental, even existential, exclusion their target populations experience” (242). Said otherwise, even equity-focused policies are ideologically bounded, materially constrained, and politically limited by structural factors related to racism, class inequity, homophobia, transphobia, and “other historically entrenched isms” that shape societies (Creary, 2021: 241).

The structures by which policies are implemented in each nation-state is another key constraint upon equity-focused policies. The 2015 federal HIV integrated planning guidance set parameters for states. It required including affected populations and stakeholders in the process, planning progress toward meeting *NHAS* goals, and the integration of programs and data infrastructures. However, because of the structure of US federalism, states have substantial leeway in implementation. This generally means that the rollout of federal programs is shaped by state-level politics and existing conditions of possibility. This was reflected in the integrated plans.

Variation across states’ approaches led us to conceptualize a “bounded justice continuum” that could account for how rigorously states’ plans addressed HIV-related equity concerns central to *NHAS* and integrated planning guidance. We then constructed a bounded justice continuum for our sample of plans using our four **RQ**s. We assigned “justice scores” of 1–5 for each of the four **RQ**s for each state. States were understood to have constructed more justice-oriented plans if they: (a) represented diverse populations in their epidemiological profiles and programmatic plans (**RQ1**); (b) stated clear goals to improve outcomes among key populations and provided concrete plans to achieve those goals by integrating programs (**RQ2**); (c) had clear plans to integrate data infrastructures (**RQ3**); and (d) engaged in greater inclusion of affected populations in their integrated planning processes (**RQ4**). A score of “1” was worst and “5” was best. States were divided among the team, scores were assigned, and then adjusted following group discussion. Each state received a final “justice score” of between a possible low of 4 and high of 20, shown in Table 4.

**Table 4.** State HIV integrated plans justice scores.

State	**RQ1** (populations)	**RQ2** (programs)	**RQ3** (data)	**RQ4** (process)	Total justice score (add **RQ1–4** scores)
Massachusetts	4	5	5	5	19
DC	4	5	5	4	18
Colorado	4	4	3	4	15
Illinois	4	4	3	4	15
Washington	4	4	3	4	15
Georgia	4	4	2	5	15
Minnesota	4	3	4	4	15
Alaska	4	4	4	3	15
New Mexico	5	4	3	3	15
California	4	5	2	3	14
New York	5	3	3	3	14
Ohio	3	3	4	3	13
Pennsylvania	4	3	2	4	13
Mississippi	2	3	3	4	12
Tennessee	3	3	4	2	12
Florida	3	3	3	3	12
Montana	2	3	3	3	11
Hawaii	2	2	3	3	10
Texas	2	2	2	2	8
Oklahoma	1	2	2	2	7

We elaborate on the bounded justice continuum below, as an overall representation of our results. This is followed by an analysis of findings under each **RQ**.

**Figure 2.**
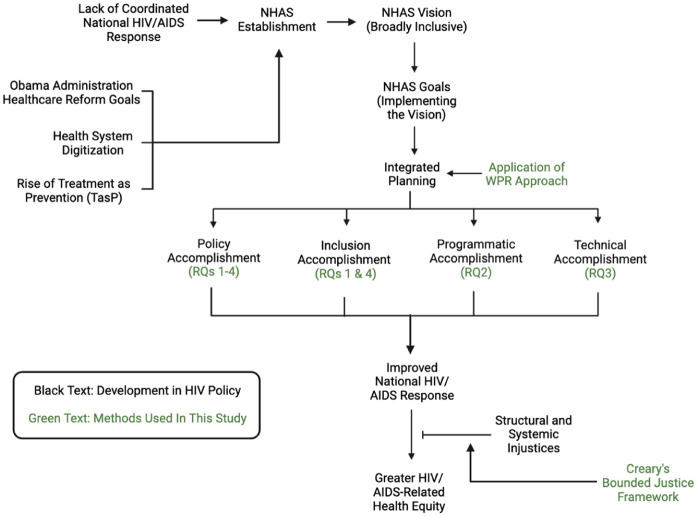
Overall analytical process and study design.

Figure 2 represents our analytical process and study design. Text in black represents a policy paradigm or problem. Text in green represents our research questions and procedures.

## Findings

### Enacting a bounded justice continuum in HIV integrated planning

Building on Creary’s (2021) “bounded justice,” we found that the HIV integrated plans and planning process created a “bounded justice continuum” whereby states worked within ideologically and materially constrained conditions to create integrated plans responsive to the equity-oriented goals of *NHAS*. The continuum accounts for how some states engaged in more robust and inclusive planning processes, and how some states produced plans more attentive to HIV-related disparities and poorly coordinated services than others.

**Figure 3.**
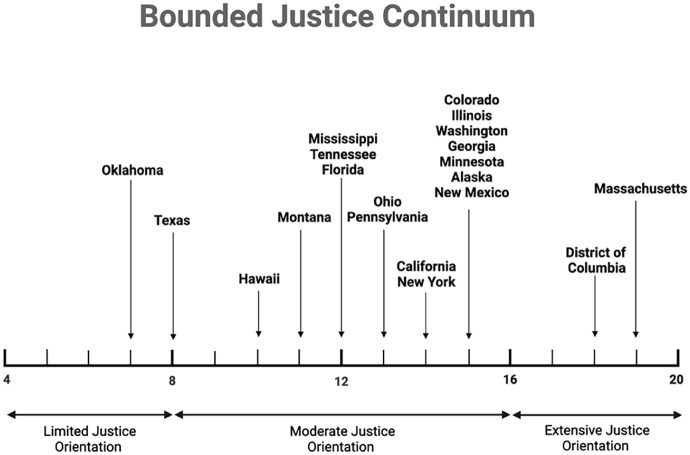
The bounded justice continuum enacted by state HIV integrated plans.

We used a definition of justice that centers inclusion as a means of pursuing social justice and health equity (Epstein, 2007). We thus treat greater inclusion of key populations and stakeholders in plans as being normatively preferable to less. Further, we treat plans that were specific and concrete in their future strategies to carry work forward as being more justice-oriented than plans which provided vague language. This enabled us to construct a normative and empirical framework that assesses the “success” of HIV integrated planning from a justice and equity-oriented perspective. Figure 3 shows where states fell on the HIV integrated planning bounded justice continuum, drawing on scores shown in Table 4.

States on the left created plans classified as less justice-oriented (i.e. less inclusive of key populations in their goals and planning processes, and without concrete plans to meet goals). Plans on the right are classified as more justice-oriented (i.e. more inclusive and with concrete plans).

### Engaging and involving stakeholders in planning processes

The federal HIV integrated planning guidance required “[t]he inclusion of community stakeholders” to “ensure that HIV prevention and care activities are responsive to the needs in the service area” (CDC and HRSA, 2015). The guidance noted the importance of including PLHIV and “representatives of varying races and ethnicities, genders, sexual orientations, ages, and other characteristics reflecting the experiences and expertise of those impacted by HIV in the jurisdiction” (15). Each plan described its community and stakeholder engagement process.

Engagement processes ranged from several meetings with advisory boards and planning groups to conducting mixed methods needs assessment studies. Every state had a stakeholder engagement strategy of some kind and at least one body with authority over planning. Some states constituted new entities for the planning process, whereas others utilized existing planning groups. For example, Georgia dissolved a previous statewide prevention planning group to create an integrated planning council, and Alaska merged two groups to form a statewide planning group. Common engagement strategies included meetings of planning groups, public comment periods, listening sessions, needs assessments, townhalls, surveys, and engaging non-governmental organizations and actors from other sectors (e.g. academia).

Two main groups of stakeholders were represented in planning processes. The first was people living with or affected by HIV, often including the demographic characteristic or groups they represented (e.g. by race/ethnicity, sexual orientation, or other characteristic like tribal membership). The second main group was representatives of different arms of the HIV sector, healthcare system, social service sectors, or non-profit ecosystem (e.g. community-based organizations, faith leaders). DC notably included former and currently incarcerated individuals. Staff from across programs were often engaged at different levels of organizational hierarchy (directors, physicians, case managers, social workers, etc.). Some states tried to make their planning process demographically representative of the state’s epidemic, such as Pennsylvania. Other states such as Minnesota, Georgia, and Oklahoma noted who was *not* involved in integrated planning to acknowledge excluded voices.

States with municipal Ryan White Part A jurisdictions covering “hard hit urban areas,” and sometimes other jurisdictional prevention planning groups, provided “letters of concurrence” or “concurrence with reservations” included in final statewide plans.^2^ Letters of concurrence from municipal HIV programs were required to attest to their participation in statewide planning processes and enabled municipalities to express satisfaction or dissent. Most letters, such as all of those in California’s integrated plan, reported full concurrence with the state’s process and final plan. However, others – such as letters in Georgia’s and Florida’s plans – expressed concurrence with reservations about aspects of the state’s HIV integrated planning process. For example, the Miami-Dade HIV/AIDS Partnership issued a concurrence with reservations to Florida’s plan because “the Partnership has only reviewed and commented on a prior version of the statewide plan and not the final version” (306). Because integrated planning ultimately fell to states, municipalities were constrained in their ability to shape the process; however, they were empowered to indicate degrees of disapproval in these letters, which were included final state integrated plans.

Some states’ plans, such as Illinois, California, and New York, were framed as “ending the epidemic” or “getting to zero” strategies, which were framings adopted by US HIV advocates during the 2010s. This represents one way that advocates’ goals were incorporated into plans, because the federal guidance did not require states to make plans centered on ending the epidemic.

### Accounting for key populations

This section describes how plans accounted for specific demographic groups. We organized our analysis by the five characteristics named in the *NHAS* vision statement: “age, gender, race/ethnicity, sexual orientation. . .or socio-economic circumstance.”^3^ We added a sixth, “intersectionality,” pertaining to how plans accounted for people who experience multiple marginalization and whether plans used the language of intersectionality.

#### Intersectionality

Intersectionality refers to how some groups experience forms of marginalization via multiple axes of oppression (Crenshaw, 1989). While intersectionality is rooted in juridical discourse and Black feminist theory, it has become increasingly central to work across many academic disciplines and health policy. Examples of intersectional populations highlighted in *NHAS* and state plans include Black gay and bisexual men; cisgender women of color; and poor gay and bisexual men of all races.

Neither *NHAS* nor federal integrated planning guidance utilized the language of intersectionality. However, priority populations were described with reference to intersecting demographic variables and risk factors, which often served as proxies for forms of oppression. For example, *NHAS* drew attention to groups that experience multiple forms of oppression, noting the “particularly high burden of HIV among. . .Black gay and bisexual men. . .young Black gay and bisexual men” and “Black transgender women” (4). These priority groups were also reflected in many state plans, particularly when emphasizing the burden among Black gay and bisexual men and transgender women, young men who have sex with men (MSM), and unhoused or unstably housed people. This tendency, reflected in plans such as Washington State and Ohio’s, shows how plans attempted to enact the equity-oriented vision of *NHAS* using an intersectional lens.

The notion that populations who experience multiple forms of marginalization merit specific programmatic attention was present throughout most plans, but to varying degrees. For example, Mississippi’s plan noted the high HIV burden among Black gay and bisexual men but did not delve into the root causes of health disparities in this group or provide robust plans to address their needs. In contrast, Tennessee, another southern state, went out of its way to address key populations affected by intersectional oppressions and described the root causes of health disparities. Tennessee noted that “HIV continues to disproportionately impact people of color – especially MSM of color – the intersection of racism, poverty, and homophobia continue to call on [Tennessee Department of Health] and its partners to renew efforts to change community norms as well as individual‘s behaviour to promote lasting impact” and that “[c]ontinued need for housing, transportation, behavioural health, and other ancillary services continue to arise as barriers” (102).

Many states attended to the social categories named in *NHAS* to describe how populations affected by HIV experienced multiple forms of oppression. These populations were represented in varying ways in narrative text, demographic tables, and descriptions of how affected populations experienced intersectional marginalization.

#### Sexuality

In *NHAS*, sexual minorities disproportionately affected by HIV were heavily emphasized, particularly gay and bisexual men. *NHAS* discussed “gay and bisexual men,” but noted that this category includes gay men, bisexual men, and MSM who do not identify as gay (19). In contrast, many state-level plans only used the behavioral classification of “MSM,” which does not account for gay/bisexual identity or the differentiation of gay and bisexual men from each other despite these being distinct sexual minority groups. *NHAS* also mentioned heterosexuals – particularly African American women – however, they did not receive the same amount of discussion as gay and bisexual men. This stratified discussion of sexual minorities was reflected in states’ plans, which tended not to focus as much on heterosexuals, owing to differences in disease burden.

The disproportionate burden of HIV on sexual minorities resulted in a heavy emphasis on sexuality across all plans. Many, such as Ohio and Mississippi, tended to utilize the MSM category. Others, such as New Mexico, opted to use the term “gay and bisexual men” in plan narratives, but “MSM” in data tables. Within the use of MSM terminology, states such as Hawaii and Oklahoma utilized MSM language, but never specifically named gay and bisexual men. This reflects a longstanding tendency in HIV programs to focus on behaviors rather than identities, which do not necessarily imply HIV risk, but also neglects to be inclusive of gay and bisexual men’s cultures (Boellstorff, 2011). Interestingly, Georgia’s prevention data had a unique category labeled “male-to-female sex with male,” representing transgender women who have sex with men. Lacking HIV prevention programs for transgender women and men who have sex with transgender women are known gaps (Poteat et al., 2021).

Plans often discussed heterosexuality using the behavioral HIV transmission category of “heterosexual contact.” Some plans included the classification “high-risk heterosexual,” although this was used inconsistently. In some plans, such as California, “high-risk heterosexuals” were defined as a category that includes both sex workers and persons who have HIV-positive sex partners. Other states merely listed high-risk heterosexuality in an inconsistently defined way or discuss heterosexuality without reference to inferred risk.

#### Sex, gender, and gender identity

*NHAS* was notable for naming “gender identity” in its vision. The strategy’s focus on transgender communities – and transgender women in particular – reflected epidemiological data about the disproportionate impact of HIV on this group and broader moves by the Obama administration to include LGBTQ populations in healthcare reform. However, *NHAS*’s attention to gender also focused on cisgender women (particularly Black women) and gay and bisexual men of all races. Therefore, in both *NHAS* and state integrated plans, discussions of gender in relation to HIV/AIDS were variably configured, including differences in how gender was reported epidemiologically and in programmatic goals.

Most states included some discussion of transgender populations. However, several, such as Oklahoma and Montana, did not report transgender data in epidemiological profiles or programmatic priorities. Some states differentiated between trans men and trans women, whereas others such as New Mexico and Texas did not break these groups into separate categories. Among plans that did discuss transgender populations, trans women were more robustly represented, partly owing to the higher rates of HIV among this population and their status as an *NHAS* priority population. Illinois’s plan notably made differential recommendations about trans men and trans women when discussing issues relevant to HIV prevention, such as healthcare personnel making often-erroneous assumptions about individuals’ anatomies or genitalia.

Lacking high-quality HIV surveillance data on transgender individuals in states was a recurrent theme. States dealt with this in a variety of ways. Texas, for example, noted the importance of including transgender people in programmatic plans while citing a lack of data. States such as Massachusetts and Georgia reported statistics about transgender people while noting that data quality needed to be improved.

#### Race and ethnicity

*NHAS* goals sought to reduce health disparities related to race and ethnicity, particularly around HIV prevalence among Black and Latino populations. While *NHAS* did address plans for reducing HIV burden for all races, there was a specific focus on Black gay and bisexual men, and Black women. These goals were reflected in state HIV integrated plans.

Across our sample, states’ HIV integrated plans noted that HIV prevalence is higher amongst racial and ethnic minority groups, particularly Black and Latino communities – especially those in the South. Data on race and ethnicity, mainly derived from HIV surveillance data, were reported across all plans, generally using standard US demographic breakdowns in tables included in their state epidemiological profiles, including White, Black/African American, Hispanic, Asian/Pacific Islander, and Native American/Alaskan Native. While the numerical data presented generally included at least these groups, discussions of data focused primarily on White, Black, and Hispanic people. Black gay and bisexual men were particularly emphasized in states in the Southeast. States without a sizable Black population, such as Alaska, still often noted the elevated burden among this population; Alaska’s plan noted that “while Blacks make up only 4% of the Alaska population, they represent 11% of reported HIV cases diagnosed in Alaska” (14).

Some states in our sample have racial/ethnic demographics that include larger Indigenous populations, including Alaska, New Mexico, Oklahoma, and Hawaii. Most of these plans contained discussions of tribal governments or other Indigenous groups, as well as specific outreach strategies and cultural competencies needed to engage, reach, and impact these communities. A notable exception to this was Oklahoma, which has the largest proportion of Native Americans of any US state; however, this population was scarcely discussed in its plan.

Some states’ plans also noted when they had large immigrant populations. Indeed, New Mexico, New York, and Washington collected data about immigrants and HIV, and their plans discuss HIV care resources available to immigrants. New Mexico, Minnesota, and New York mentioned the difficulty of providing HIV care to undocumented persons. Migrant populations were absent from the Texas plan despite the state’s large immigrant population, a fact likely attributable to anti-immigrant politics in Texas. Minnesota’s plan mentioned the ability of undocumented PLHIV to receive care at Federally Qualified Health Centers.

#### Class or socio-economic circumstance

The critical influence of income and social class on HIV-related health disparities and care outcomes was illustrated by discussions of class and socioeconomic circumstance across states’ plans. Unlike HIV prevalence, race/ethnicity data, and data about retention in care, income statistics are not collected as part of routine HIV surveillance. So, integrated planning entities turned to various other data instruments and proxy measures to discuss the impact of class inequity on PLHIV in their states. Some available data sources included Medicaid enrollment and utilization statistics, Ryan White data, and housing databases. The reliance on non-standardized measures for income across plans led to states addressing the impact of class differently.

For example, in programmatic language, Oklahoma and Texas acknowledged poverty as a barrier to access, but their plans lacked specific actionable goals related to poverty. Conversely, New York, Washington, Tennessee, and Montana offered detailed discussions on the financial hurdles faced by PLHIV in their states, with Tennessee further recognizing the limited care options due to the absence of Medicaid expansion. These states developed detailed goals for addressing highlighted proxies of poverty such as increasing Medicaid enrollment eligibility and transitioning PLHIV from Ryan White services (which only covers HIV-related care) onto more comprehensive ACA insurance policies. Additionally, Medicaid expansion greatly shaped discussions of class within the plans. States that expanded Medicaid often provided a more detailed analysis of financial barriers and solutions. States that opted not to expand Medicaid had less available funding for healthcare and supportive services and were constrained in their ability to use insurance enrollment as a strategy for improving HIV-related health equity.

Housing was often used as a proxy measure for low-income status. However, discussions of housing were often broad and focused on access and inequity rather than providing plans to address housing-related disparities. Some states such as Illinois discussed the Housing Opportunities for Persons with AIDS program. However, plans’ primary focus remained on allocating resources for prevention and care rather than addressing social housing needs. This perspective on housing access and equity reflects the restricted positions of planners, whose primary objective was to allocate HIV resources rather than solve deep-seated housing issues. Consequently, while housing was recognized as critical to improving outcomes, housing-related solutions in plans were often limited by the scope and goals of HIV integrated planning.

#### Age

*NHAS* discussed age throughout the document and included “[y]outh aged 13 to 24 years” as a key population. This focus was present in almost all state integrated plans and could be seen first in the epidemiologic profiles. There was often less stratification for older people, who were grouped together in tables typically reporting 50 or 60 years and older. This relatively low age cutoff for data stratification is of particular interest, because approximately 70% of PLHIV will be over the age of 50 by 2030 (Wing, 2016).

Beyond state epidemiological profiles, most plans centered young PLHIV when exploring barriers to care or setting goals and activities. Long-term survivors or individuals over 65 were less discussed. Medicare enrollment numbers sometimes served as a proxy for older PLHIV.

However, some plans, such as DC’s, had a more robust discussion on long-term survivors and newly diagnosed older adults. Other states indirectly touched on challenges faced by aging PLHIV by discussing Medicare coverage and healthcare access. Of note, Pennsylvania and DC discussed the growing need for HIV providers and services targeting aging PLHIV, showing stronger awareness of the increasing complexity of care for this group.

Interestingly, intersectional identities were robustly represented in relation to young PLHIV, and communities at the intersection of age, race, and sexual orientation were consistently emphasized. Young MSM of color, in particular, were consistently highlighted, due to emphasis of this population in *NHAS* and higher incidence of HIV in young individuals and people of color.

State plans also presented a variety of methods for goal operationalization regarding specific age groups. For example, despite Montana’s plan having little discussion regarding age, the goals section provided activities targeted toward young MSM of color. New Mexico similarly discussed challenges faced by both young and aging PLHIV but scarcely represented these groups in activities and goals. This highlighted the difference between states that focused on descriptions of age and HIV and those that emphasized programmatic change to improve health equity across age groups.

Overall, youth were heavily emphasized in integrated plans. Despite older adults growing as a proportion of PLHIV, this age group was far less emphasized. States’ goal planning and resource allocation showed a concerted effort to stem new HIV cases in young groups, which were an *NHAS* priority population.

### Programmatic integration

One key goal of federal HIV integrated planning was to integrate programs across the domains of HIV care, surveillance, and prevention, which have historically operated somewhat separately from one another under different funding streams. The rationale for doing so was to help realize the potential benefits of treatment-as-prevention as a public health strategy. States undertook varied approaches for integrating different arms of the HIV public health sector and services with other arms of the social safety net, such as sexually transmitted disease (STD) programs and housing and nutritional support. The overall goals of programmatic integration were to help PLHIV become retained in long-term care.

Many states’ plans emphasized improved coordination across sectors to improve linkage-to-care among people newly diagnosed or who were vulnerable to falling out of care. Georgia and Tennessee’s plans detailed linkage to care initiatives in partnership with correctional facilities that aimed to link PLHIV to care upon release. These efforts linked the goals of integrating arms of the state that provide services to states’ viral suppression targets. Washington State aimed to increase “health care navigation” for people living with and affected by HIV and to integrate the work of STD and HIV field services, providing “a wrap-around health service, which helps to improve health through detection, treatment, and prevention of disease” (32). These initiatives sought to improve rates of new diagnosis, linkage, and retention by working across multiple parts of the health system.

Programmatic integration was less of a focus in other states’ plan. For example, Oklahoma did not provide detailed plans about how HIV programs would be integrated, but did note that a task force had formed and that a new linkage-to-care coordinator position was created. Other states, like Montana, asserted that programmatic integration had already been achieved because HIV services and/or relevant programs (e.g. STD, hepatitis) were housed together at the state health department.

### Integrating data systems

*NHAS*, federal integrated planning guidance, and related HIV programs set forth goals to strengthen and integrate HIV data systems across the domains of care, prevention, and surveillance (Molldrem and Smith, 2020). Data interoperability was pursued to help improve linkage-to-care and resource utilization across programs, and also to more fully understand epidemics by integrating datasets that were previously relatively “siloed” (Cohen et al., 2014; Molldrem, 2020; Satcher Johnson et al., 2024). Because data integration is linked with programmatic integration and monitoring, these initiatives were often woven throughout different parts of plans. Plans ranged from having a section dedicated to this topic, to data integration being incorporated into states’ programmatic goals or appendices.

A wide spectrum of data integration methods was proposed. Oklahoma’s plan, for example, had little data integration discussion beyond a few statements that affirmed “complete sharing of data” within the state health department (32). Others, such as DC and Georgia, had more concrete plans regarding how integration would be achieved, and which organizations and activities would be involved. States such as Illinois, Georgia, Pennsylvania, and Minnesota reflected on data sources that they would have liked access to in the planning process but did not, including private insurance and Medicare data. For states with more robust data integration plans, methods ranged from improving re-linkage-to-care programs to targeting organizations with inaccessible data for integration.

States’ plans also displayed varying degrees of actualized data integration. Alaska’s plan claimed that its HIV/STD Program was already “fully integrated” and that “there are no data policies that prohibit sharing of data between HIV Surveillance, HIV Care, and HIV Prevention” (82). On the other hand, Montana seemed to lack full data integration, as multiple data sets were used to analyze different population metrics, resulting in a goal to implement a re-linkage program using HIV surveillance data by 2018. Other states provided greater reflection on the current state of their data systems. For example, Hawaii described its current data sources and their respective strengths and weaknesses.

In general, states with stronger data system integration reported more in-depth planning to programmatically actualize data integration. Overall, most states had made previous progress toward data integration and formed plans for data system integration and expansion of re-linkage programs or similar programs that utilized HIV surveillance data to identify PLHIV who had fallen out of care and then reach out to them.

Several plans described how they had to work among walled-off data systems, such as private insurance companies, and within systemic constraints – such as data policies that restricted exchange between organizations – to make piecemeal additions and agreements to integrate HIV data systems. Restrictions on data sharing between surveillance and prevention programs further limited sharing efforts and was framed as hindering *NHAS*’s goal of increased linkage and retention.

## Discussion

We analyzed 20 state HIV integrated plans created under 2015 federal guidance. We sought to understand how integrated planning was pursued as part of realizing *NHAS*’s health equity-focused vision. We used Bacchi’s WPR approach and Creary’s concept of “bounded justice” to examine how states’ integrated plans accounted for justice considerations and populations disproportionately affected by HIV named in *NHAS*’s vision. We placed our sampled jurisdictions along a “bounded justice continuum” representing their plans’ incorporation of justice- and equity-oriented goals. We found that states all worked to integrate *NHAS*’s vision, but to varying degrees that represented differing political situations in states, infrastructural issues such as the ability to share data, and material constraints related to the funding of social services.

The bounded justice continuum contributes to the literature on bounded justice and health equity by placing the process of policy implementation by jurisdictional actors at the center of studying how bounded justice paradigms are actualized (Creary, 2021). The concept can be useful for researchers seeking to understand how policy frameworks that establish parameters for achieving justice in federal systems such as those in the US can lead to differential implementations and outcomes which reflect both the priorities of higher-order (i.e. federal) actors and lower-order (i.e. state and local) ones. Future studies of how bounded justice continuums are enacted in the implementation of policy regimes in federal systems could utilize multi-sited ethnographic fieldwork, document analysis, or other approaches that comparatively explore local variations in the implementation of justice and equity-oriented policies set by national actors. The structure of our research questions and our empirical operationalization of the bounded justice continuum could also potentially be useful for researchers to incorporate into efforts such as health equity impact assessments, particularly when assessing initiatives implemented in federated systems (Olyaeemanesh et al., 2023).

We adopted a notion of justice that prioritized inclusion of populations and the integration of programs and systems, taking the position that the integration of HIV programs and data systems would contribute to more socially just outcomes. We did so on the basis that, if ethically implemented, these programs could theoretically improve outcomes (Satcher Johnson et al., 2024). However, we note that there is not a consensus that the integration of HIV programs and data systems advance social justice (Dennis et al., 2023; Molldrem and Smith, 2020). Debates about this continue, related mainly to issues concerning privacy, confidentiality, consent, and community and stakeholder engagement (Bollinger et al., 2023; Molldrem et al., 2025; Molldrem and Smith, 2020).

The bounded justice continuum attests to the fact that some states were better able to address *NHAS*’s equity-focused vision than others. Our findings also affirm the basic fact that neither HIV integrated planning nor any single policy paradigm can solve the entrenched social inequities that drive HIV/AIDS epidemics. HIV/AIDS epidemics are sustained by a lack of economic opportunity rooted in capitalist exploitation, systemic racism rooted in racial oppression, homophobia, transphobia, anti-immigrant sentiment stoked by the far right, and related systems of oppression (Geary, 2014). The WPR approach helps to describe how policy regimes attempt to represent social problems and state strategies for managing them, and Creary’s bounded justice framework is designed to account for how equity-oriented actors work to achieve more socially just outcomes within oppressive structures. Therefore, while integrated planning ultimately appeared to contribute to a slightly more equitable HIV response, this could only amount to marginal improvement. Integrated planning and programmatic integration cannot address issues such as lacking funding for healthcare. Many plans spoke about this, particularly regarding lacking Medicaid expansion and housing. These ongoing issues speak to the need for transformational policies that can address the root causes that drive HIV/AIDS epidemics by ensuring social benefits such as free universal healthcare, guaranteed housing, supportive services, and employment.

Our study has several limitations. We only analyzed a subset of 20 plans, and we only focused on plan text. However, we created a balanced sample by accounting for key factors such as region, rural/urban geography, racial/ethnic demographics, Medicaid expansion, and varied HIV epidemics. The WPR approach and bounded justice framework also have limitations. By focusing on how specific problems are constituted in policy, WPR can create analytical blind spots. However, WPR accounts for this by explicitly centering the main framing of a policy problem and potential alternatives. Bounded justice, in turn, focuses more on barriers to achieving justice than on actualizing deep structural transformation. By combining WPR with bounded justice, we aimed to draw on these approaches’ strengths in order to account for constraints upon integrated plans’ ability to achieve just outcomes.

Future research could examine the second round of HIV integrated plans created beginning in 2022 under revised guidance. The period between 2015 and 2017 (when the plans we analyzed were created) to 2022 saw the release of the national *Ending the HIV Epidemic Initiative* in 2019, but only marginal improvements in the national HIV response. Indeed, the goal of “ending the epidemic” by 2030 appearing increasingly unachievable (Sullivan et al., 2021). Other studies could include empirical analyses of HIV integrated planning using ethnography or other qualitative methods.

We found that HIV integrated planning was differentially enacted by states under the equity-oriented vision of *NHAS*, which sought to address the problem of an inequitable and poorly coordinated national response. This created a bounded justice continuum wherein some states used the opportunity to enact integrated plans that more robustly addressed the problems of equity and justice. Some states, such as Georgia, advanced equity in their plans even while working in unfavorable political conditions where Medicaid had not been expanded. Others, such as Massachusetts, leveraged robust public health infrastructures and existing HIV initiatives as part of their planning. However, in no state was integrated planning a panacea for the deep-rooted inequities that forestall progress toward ending the US HIV/AIDS epidemic. Until transformational social reforms are passed to ensure universal access to care, supportive services, and basic needs like housing and employment, frameworks such as integrated planning will only be able to marginally improve the country’s HIV response.

## Notes

1. *NHAS* was released in 2010. However, we cite *NHAS: Updated to 2020*, released in 2015, because it was the version in place as states undertook integrated planning from 2015 to 2017 (ONAP, 2015).

2. The Ryan White program is payer of last resort for HIV-related medical care in the US.

3. The *NHAS* vision statement listed “gender” and “gender identity” separately; we collapse this into the single category of “gender,” because “gender identity” is a component of gender.

## References

[bibr1-13634593251375041] Bacchi C (2012) Introducing the ‘What’s the problem represented to be?’ approach. In: Bletsas A Beasley C (eds) Engaging With Carol Bacchi. Strategic Interventions and Exchanges. University of Adelaide Press, pp.21–24. Available at: http://www.jstor.org/stable/10.20851/j.ctt1sq5x83.7 (accessed 11 Ocober 2023).

[bibr2-13634593251375041] Bacchi CL Goodwin S (2016) Poststructural Policy Analysis: A Guide to Practice. Palgrave Pivot. Palgrave Macmillan.

[bibr3-13634593251375041] Boellstorff T (2011) But do not identify as gay: A proleptic genealogy of the MSM category. Cultural Anthropology 26(2): 287–312.

[bibr4-13634593251375041] Bollinger JM Geller G May E , et al. (2023) Brief report: Challenges in obtaining the informed perspectives of stakeholders regarding HIV molecular epidemiology. JAIDS Journal of Acquired Immune Deficiency Syndromes 93: 87–91. 36805407 10.1097/QAI.0000000000003179 PMC10293093

[bibr5-13634593251375041] Centers for Disease Control and Prevention (CDC) and Health Resources and Services Administration (HRSA) (2015) Integrated HIV Prevention and Care Plan Guidance, including the Statewide Coordinated Statement of Need. U.S. Department of Health and Human Services. Available at: https://web.archive.org/web/20241211074646/https://ryanwhite.hrsa.gov/sites/default/files/ryanwhite/grants/hiv-prevention-plan-06-2015.pdf (accessed 23 August 2023).

[bibr6-13634593251375041] Cohen SM Gray KM Ocfemia MCB , et al. (2014) The status of the National HIV Surveillance System, United States, 2013. Public Health Reports 129(4): 335–341. 24982536 10.1177/003335491412900408 PMC4037459

[bibr7-13634593251375041] Creary MS (2021) Bounded justice and the limits of health equity. Journal of Law Medicine & Ethics 49(2): 241–256. 10.1017/jme.2021.34 PMC8245211 34924041

[bibr8-13634593251375041] Crenshaw K (1989) Demarginalizing the intersection of race and sex: A Black Feminist Critique of Antidiscrimination Doctrine, Feminist Theory and antiracist politics. University of Chicago Legal Forum 1989: 139–167.

[bibr9-13634593251375041] Dawes DE (2016) 150 Years of ObamaCare. Johns Hopkins University Press.

[bibr10-13634593251375041] Dennis A Day S Rennie S , et al. (2023) Revitalizing Community Engagement in the Public Health Use of Molecular HIV Epidemiology. University of North Carolina, Chapel Hill. Available at: https://dennis.unc.edu/wp-content/uploads/sites/3288/2023/04/MHE_WhitePaper_2023-04-28.pdf (accessed 4 July 2024).

[bibr11-13634593251375041] Epstein S (2007) Inclusion: The Politics of Difference in Medical Research. University of Chicago Press.

[bibr12-13634593251375041] Fairchild AL Bayer R (2011) HIV surveillance, public health, and clinical medicine — Will the walls come tumbling down? New England Journal of Medicine 365(8): 685–687. 21864165 10.1056/NEJMp1107294

[bibr13-13634593251375041] Fairchild AL Bayer R Colgrove JK , et al. (2007) Searching Eyes: Privacy, the State, and Disease Surveillance in America. California/Milbank Books on Health and the Public 18. University of California Press; Milbank Memorial Fund.

[bibr14-13634593251375041] Geary AM (2014) Antiblack Racism and the AIDS Epidemic: State Intimacies. Palgrave Macmillan.

[bibr15-13634593251375041] Kaiser Family Foundation (2025) HIV Viral Suppression Rate in U.S. Lowest Among Comparable High-Income Countries. Kaiser Family Foundation. Available at: https://web.archive.org/web/20251008151655/https://www.kff.org/hiv-aids/hiv-viral-suppression-rate-in-u-s-lowest-among-comparable-high-income-countries/ (accessed 8 October 2025).

[bibr16-13634593251375041] McCray E Mermin J (2017) ‘Dear Colleague’ letter announcing CDC’s position that ‘people who take ART daily as prescribed and achieve and maintain an undetectable viral load have effectively no risk of sexually transmitting the virus to an HIV-negative partner.’ Dear Colleague Letter. Available at: https://web.archive.org/web/20171102063511/https://www.cdc.gov/hiv/library/dcl/dcl/092717.html (accessed 11 October 2023).

[bibr17-13634593251375041] Molldrem S (2020) How to build an HIV out of care watch list: Remaking HIV surveillance in the era of treatment as prevention. First Monday 25(10): 20.

[bibr18-13634593251375041] Molldrem S Smith AKJ (2020) Reassessing the ethics of molecular HIV surveillance in the era of cluster detection and response: Toward HIV data justice. American Journal of Bioethics 20(10): 10–23. 10.1080/15265161.2020.1806373 32945756

[bibr19-13634593251375041] Molldrem S Smith AKJ Chung C , et al. (2025) HIV data and public health ethics. Public Health Ethics 18(3): af008.

[bibr20-13634593251375041] Olyaeemanesh A Takian A Mostafavi H , et al. (2023) Health Equity Impact Assessment (HEIA) reporting tool: Developing a checklist for policymakers. International Journal for Equity in Health 22(1): 241. 37980523 10.1186/s12939-023-02031-0 PMC10657117

[bibr21-13634593251375041] ONAP and White House Office of National AIDS Policy (2015) The National HIV/AIDS Strategy for the United States: Updated to 2020. The White House.

[bibr22-13634593251375041] ONC (2015) Connecting Health and Care for the Nation: A Shared Nationwide Interoperability Roadmap Final Version 1.0. The Office of the National Coordinator for Health Information Technology (ONC). Available at: https://www.healthit.gov/sites/default/files/hie-interoperability/nationwide-interoperability-roadmap-final-version-1.0.pdf. (accessed 11 October 2023).

[bibr23-13634593251375041] Poteat T Cooney E Malik M , et al. (2021) HIV prevention among cisgender men who have sex with transgender women. AIDS and Behavior 25(8): 2325–2335. 33634354 10.1007/s10461-021-03194-z PMC8222096

[bibr24-13634593251375041] Rodger AJ Cambiano V Bruun T , et al. (2016) Sexual activity without condoms and risk of HIV transmission in serodifferent couples when the HIV-positive partner is using suppressive antiretroviral therapy. JAMA 316(2): 171. 27404185 10.1001/jama.2016.5148

[bibr25-13634593251375041] Satcher Johnson A Peruski A Oster AM , et al. (2024) Enhancements to the National HIV Surveillance System, United States, 2013-2023. Public Health Reports 139: 654–661. 38822672 10.1177/00333549241253092 PMC11528829

[bibr26-13634593251375041] Stewart-Winter T (2018) The Gay Rights President. In: Zelizer J (ed.) The Presidency of Barack Obama. Princeton University Press, pp.95–110. Available at: https://www.degruyter.com/document/doi/10.23943/9781400889556-009/html. (accessed 11 October 2023).

[bibr27-13634593251375041] Sullivan PS Satcher Johnson A Pembleton ES , et al. (2021) Epidemiology of HIV in the USA: Epidemic burden, inequities, contexts, and responses. Lancet 397(10279): 1095–1106. 33617774 10.1016/S0140-6736(21)00395-0 PMC12707558

[bibr28-13634593251375041] Sweeney P Gardner LI Buchacz KATE , et al. (2013) Shifting the paradigm: Using HIV surveillance data as a foundation for improving HIV care and preventing HIV infection: Using HIV surveillance data to improve care and prevent infection. Milbank Quarterly 91(3): 558–603. 24028699 10.1111/milq.12018 PMC3790525

[bibr29-13634593251375041] The White House (2015) FACT SHEET: The National HIV/AIDS strategy: Updated to 2020. Available at: https://web.archive.org/web/20250304000102/https://obamawhitehouse.archives.gov/the-press-office/2015/07/30/fact-sheet-national-hivaids-strategy-updated-2020 (accessed 18 March 2025)

[bibr30-13634593251375041] Wing EJ (2016) HIV and aging. International Journal of Infectious Diseases 53: 61–68. 27756678 10.1016/j.ijid.2016.10.004

[bibr31-13634593251375041] Yehia B Frank I (2011) Battling AIDS in America: An evaluation of the National HIV/AIDS strategy. American Journal of Public Health 101(9): e4–e8. 10.2105/AJPH.2011.300259 PMC3154226 21778507

